# A trait–environment relationship approach to participatory plant breeding for organic agriculture

**DOI:** 10.1111/nph.18203

**Published:** 2022-05-24

**Authors:** Andrés G. Rolhauser, Emma Windfeld, Solveig Hanson, Hannah Wittman, Chris Thoreau, Alexandra Lyon, Marney E. Isaac

**Affiliations:** ^1^ Department of Physical and Environmental Sciences University of Toronto Scarborough Toronto ON M1C 1A4 Canada; ^2^ Departamento de Métodos Cuantitativos y Sistemas de Información Facultad de Agronomía Universidad de Buenos Aires Buenos Aires C1417DSE Argentina; ^3^ Facultad de Agronomía IFEVA Universidad de Buenos Aires CONICET Buenos Aires C1417DSE Argentina; ^4^ Department of Geography University of Toronto Toronto ON M5S 3G3 Canada; ^5^ School of Public Policy Simpson Centre University of Calgary Calgary AB T2P 1H9 Canada; ^6^ Center for Sustainable Food Systems University of British Columbia Vancouver BC V6T 1Z2 Canada; ^7^ Department of Sustainable Agriculture and Food Systems Kwantlen Polytechnic University Richmond BC V6X 3X7 Canada

**Keywords:** crop performance, *Daucus carota*, functional traits, leaf traits, linear mixed model, root traits, selection, soil nutrients

## Abstract

- The extent of intraspecific variation in trait–environment relationships is an open question with limited empirical support in crops. In organic agriculture, with high environmental heterogeneity, this knowledge could guide breeding programs to optimize crop attributes. We propose a three‐dimensional framework involving crop performance, crop traits, and environmental axes to uncover the multidimensionality of trait–environment relationships within a crop.
- We modeled instantaneous photosynthesis (*A*_sat_) and water‐use efficiency (WUE) as functions of four phenotypic traits, three soil variables, five carrot (*Daucus carota*) varieties, and their interactions in a national participatory plant breeding program involving a suite of farms across Canada. We used these interactions to describe the resulting 12 trait–environment relationships across varieties.
- We found one significant trait–environment relationship for *A*_sat_ (taproot tissue density–soil phosphorus), which was consistent across varieties. For WUE, we found that three relationships (petiole diameter–soil nitrogen, petiole diameter–soil phosphorus, and leaf area–soil phosphorus) varied significantly across varieties. As a result, WUE was maximized by different combinations of trait values and soil conditions depending on the variety.
- Our three‐dimensional framework supports the identification of functional traits behind the differential responses of crop varieties to environmental variation and thus guides breeding programs to optimize crop attributes from an eco‐evolutionary perspective.

## Introduction

Functional phenotypic traits mediate the response of plants to environmental conditions, providing a mechanistic link between environmental change and community dynamics (Lavorel & Garnier, 2002; Violle *et al*., 2007). Understanding this link is crucial for predicting future plant responses to global change (Thuiller *et al*., 2008; Scheiter *et al*., 2013). In theory, traits relate to environmental gradients via fundamental eco‐physiological principles that lead to general trait–environment relationships (Westoby *et al*., 2002; Garnier *et al*., 2016). However, empirical evidence shows differences in trait–environment relationships across communities and regions (Westoby *et al*., 2002; Garnier *et al*., 2016; Funk *et al*., 2017) and also across species found in a region (Kichenin *et al*., 2013; Lajoie & Vellend, 2015; Dong *et al*., 2020; Buchanan *et al*., 2021; Sarker *et al*., 2021). This leads to the question of whether trait–environment relationships also vary between genotypes of the same species, for which specific empirical tests are lacking (Westerband *et al*., 2021). Well‐described intraspecific trait–environment relationships would improve our capacity to make accurate predictions about the performance and fate of particular target species, such as endangered species, weeds, and importantly, crops. For crops in particular, it will help guide selection methods and breeding programs to optimize crop attributes from an eco‐evolutionary perspective.

**Fig. 1:**
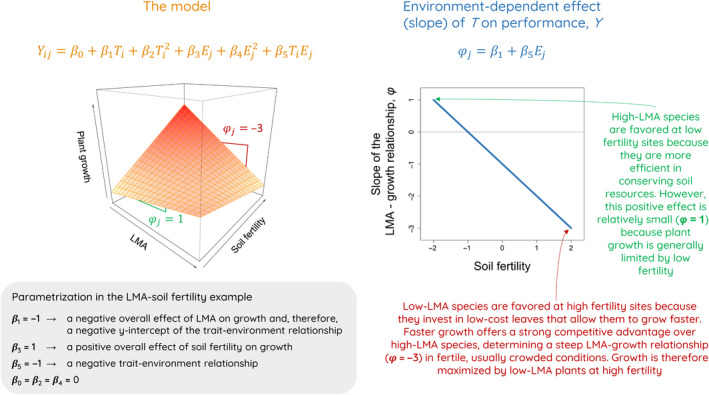
Example of the three‐dimensional approach to trait–environment relationships. Generically, plant performance (*Y*) is modeled as a function of the interaction between trait (*T*) and environmental (*E*) axes. Our model (orange equation above three‐dimensional plot; see details in the Materials and Methods section) also includes quadratic terms for both *T* and *E*, set to zero here for simplicity. Parameters *β*_1_ and *β*_5_ control the relationship between *T* and *E*, modeled as an environment‐dependent effect of *T* on *Y* (denoted *φ*). In this example, plant growth is a function of leaf mass per area (LMA) and soil fertility – we expect that high soil fertility selects for acquisitive, low‐LMA phenotypes. Using a simple parametrization (gray box), the predicted response surface (left plot) is an asymmetric saddle with a peak at low LMA and high soil fertility. This results in a negative LMA–soil fertility relationship (right plot) that is shifted downwards with respect to zero (dotted line) due to the overall negative effect of LMA on growth.

Most research on trait–environment relationships use direct regressions of trait values against environmental variables, i.e. trait–environment regressions. This approach assumes, among other things, that traits are directly (causally) affected by environmental conditions. However, trait–environment relationships result from selection – whether by natural or human‐facilitated processes – that is, when environmental conditions drive a specific trait expression over another depending on the fitness of such trait values (Shipley, 2010; Laughlin & Messier, 2015; Shipley *et al*., 2016; Vellend, 2016). In a three‐dimensional space, environmentally‐dependent selection emerges as the slope of trait–fitness relationships changing along an environmental gradient, i.e. a trait–environment interaction effect on fitness (Fig. 1). Following McGill *et al*. (2006), ‘performance currencies’ substitute fitness, as eco‐physiological measures related to the acquisition and allocation of energy and nutrients, which are thought to be closely connected to the physical environment. More specifically, a performance currency (hereafter ‘performance’ for simplicity) affects fitness via vital rates (growth, survival, fecundity), in a trait–performance–vital rate–fitness causal chain (Geber & Griffen, 2003).

Studies on crop response to environmental conditions are ubiquitous in the plant breeding literature and often seek to identify genotype × environment interactions (Allard & Bradshaw, 1964; e.g. Seljåsen *et al*., 2012; Ulrich *et al*., 2015, in carrot) or apply Hildebrand & Russell's (1996) adaptability analysis framework to identify crop varieties with either broad or specific environmental adaptation (e.g. Lyon *et al*., 2020, in carrot and other crops). In contrast, studies on crop trait–environment relationships are rare and have been based on trait–environment regressions (e.g. Gagliardi *et al*., 2015; Martin *et al*., 2018). This reflects the current paradigm of trait‐based agroecology, which conceives of phenotypic traits as directly determined by local environmental conditions, while assuming that observed patterns would be the result of selection and adaptation (Garnier *et al*., 2016; Damour *et al*., 2018; Martin & Isaac, 2018). This approach has been valuable in describing patterns of crop intraspecific trait variation, but application of the three‐dimensional framework described earlier would extend the trait‐based agroecological approach to uncover performance‐based selective processes. In particular, it is important to evaluate whether crop trait–environment relationships are the result of general, species‐wide phenotypic plasticity or whether different crop genotypes display different trait–environment relationships when predicting performance. This information would be vital to predict the adaptability of different crop genotypes not only under transitions to organic agricultural systems but also in the face of environmental change.

Here we focus on how trait–environment relationships differ among crop varieties using a participatory network of Canadian organic farms and *Daucus carota* subsp. *sativus* (cultivated carrot; hereafter carrot) as a model species. Participatory plant breeding (PPB) is a crop development strategy that facilitates farmer‐involved selection in environments that diverge from those targeted by conventional plant breeding (Atlin *et al*., 2001). PPB was first developed to serve farmers cultivating marginal land (Ceccarelli, 1994), but more recently, PPB has been adapted to serve organic, diversified, and lower‐input farms in the Global North (e.g. Mazourek *et al*., 2009; Shelton & Tracy, 2015). Conventional agriculture relies on high levels of synthetic inputs and leads to landscape simplification and homogenization (Tscharntke *et al*., 2005). Conversely, the use of organic amendments often leads to higher spatial and temporal variability in soil nutrients, underscoring high environmental heterogeneity in these agroecosystems (Isaac *et al*., 2021). Therefore, breeding goals in PPB are two‐fold: generate specific adaptations to stressful, but predictable growing conditions (such as poor soil conditions), and promote performance stability in the face of temporal environmental variability (Ceccarelli, 1994; Dawson *et al*., 2008).

In general, yield in crop species with vegetative storage, such as carrot, is not usually limited by sink demand, since storage cells in existing organs can be continuously formed, adjusting sink capacity to current photosynthate supply (Engels *et al*., 2012). Improving photosynthetic capacity is therefore a major goal in breeding programs of source‐limited crops (Flood *et al*., 2011). Carrots prefer light‐textured soils with low moisture retention, thus requiring frequent irrigation to achieve high yields and good market quality (Rubatzky *et al*., 1999). Therefore, improving water‐use efficiency (WUE) in irrigation‐requiring crops is important given increasing freshwater demand (De Pascale *et al*., 2011). In addition, breeding for plasticity in WUE could lead to better survival and higher average yields in the face of increased climate variability and climate extremes (Nicotra *et al*., 2010). The three‐dimensional functional trait analysis is therefore particularly relevant for PPB and carrots because it provides a mechanistic understanding of crop performance variation across a range of environmental conditions.

We evaluated the effect of trait–soil interactions on two measures of plant performance in carrot: instantaneous photosynthesis (*A*_sat_) and instantaneous photosynthetic WUE (the ratio between *A*_sat_ and transpiration). We modeled *A*_sat_ and WUE as a function of four morphological traits and three soil properties. Traits were leaf area (LA), leaf mass per area (LMA), petiole diameter (PD), and taproot tissue density (TTD). While these traits are not commonly measured in carrot breeding studies, they are relatively easy to measure and have been identified as drivers of plant performance in the broader ecological literature. Leaf mass per area represents a trade‐off between leaf longevity and resource conservation, maximized at high LMA, and potential growth rate, maximized by low LMA (Westoby *et al*., 2002; Poorter *et al*., 2009). Similarly, root tissue density represents a belowground trade‐off between resource conservation and potential growth (Weemstra *et al*., 2016), while TTD is specific for taproots (Fort *et al*., 2015). Taproot tissue density may also relate to carrot texture, important for sensory quality (Paoletti *et al*., 2012). Leaf area and petiole diameter are size traits that are allometrically related (Price & Enquist, 2007). Leaf area represents, at least in part, a trade‐off between light interception efficiency via lower self‐shading and heat dissipation via gas exchange (Givnish, 1987; Westoby *et al*., 2002). In crops, leaf size has been identified as an important trait improving competitive ability against weeds, particularly at early growth phases (Andrew *et al*., 2015). Petioles provide mechanical and hydraulic support to the lamina (Niinemets *et al*., 2007; Poorter & Rozendaal, 2008), and PD partially captures this functionality (Anten *et al*., 2010). For carrot in particular, petioles are agronomically important given their role in mechanical harvesting of roots (Rogers & Stevenson, 2006; Turner *et al*., 2018). In general, we expect that plant acquisitive trait expression (lower LMA and TTD, and higher LA and PD) would be associated with higher *A*_sat_ and lower WUE. However, importantly, our three‐dimensional framework underscores that trait–performance relationships are not fixed but environment‐dependent, meaning that trait–performance relationships may change along a sufficiently long environmental gradient (as in Fig. 1; see also Laughlin *et al*., 2018).

Soil nitrogen (N), phosphorus (P), and carbon (C) were selected as soil properties particularly indicative of soil fertility in organic agriculture, given that organic amendments are a primary source of nutrients for crops (Drinkwater & Snapp, 2007). Further, total soil C (and soil organic matter) has direct and indirect effects on soil structure, soil moisture, and plant‐available nutrients (Chen *et al*., 2018). Such beneficial soil conditions can enhance photosynthesis, and, to a lesser degree, transpiration, thus also increasing WUE (Raven *et al*., 2004). On this basis, we generally expect favorable soil conditions for plant growth (high N, P, and C) to select for acquisitive plant strategies characterized by low LMA and TTD, and high LA and PD when performance is measured as *A*_sat_ (see prediction example in Fig. 1). We also expect steeper trait–environment relationships when performance is measured as WUE compared to *A*_sat_, since beneficial soil conditions affect photosynthesis more strongly than transpiration.

## Materials and Methods

### Experimental design

Cultivated carrot, a diploid (2*n* = 18) outcrossing biennial crop, was domesticated from wild carrot *D. carota* subsp. *carota,* or Queen Anne’s Lace (Ellison, 2019). Domesticated carrot is classified phenotypically and genotypically into Eastern and Western types (Grzebelus *et al*., 2014). Within the Western cultivar type, market classes are defined on the basis of root shape and culinary use (Luby *et al*., 2016). We focused on Western cultivars of the Nantes market class based on farmer‐participant preference; Nantes cultivars are characterized by blunt tips, minimal tapering, and good storage ability.

Nine farmers were recruited from the Canadian Organic Vegetable Improvement (CANOVI) project, an established network of farmers across Canada (see map of participating farms in Supporting Information Fig. S1). CANOVI project farmers have been trialing Nantes‐type carrot varieties as part of a national PPB program facilitated by the University of British Columbia and the Bauta Family Initiative on Canadian Seed Security. The nine selected farm sites spanned an environmental gradient across Canada (Table S1). Across the nine farms, average monthly temperatures for the June–August growth period ranged from 16.6 to 20.5°C, while average total precipitation ranged from 97.2 to 237.9 mm across the farms (Table S1). Seasonal growing degree days (GDD) ranged from 1425 to 2329.5 GDD above 5°C (Table S1). Uniform management instructions were provided to all farms, specifying the use of organic pest and disease control measures, and allowing flame weeding.

At each farm, in 2019, five Nantes‐type carrot varieties were planted, each one in a single plot measuring a minimum of 2 m with no < 25 cm between‐row spacing. These comprised two hybrid varieties considered industry‐wide standard varieties (‘Bolero’ and ‘Naval’) from different continental European breeding programs; two open pollinated varieties bred by a farmer‐breeder in the Pacific Northwest (‘Rumba’ and ‘Nash’s Nantes’); and one open pollinated variety bred in Switzerland and introduced to the North American market for its performance on Northern Atlantic farms (‘Dolciva’). Both hybrid varieties were bred under conventional field conditions, while all three open pollinated varieties were bred in organic conditions; variety introduction dates, breeders, and seed sources are detailed in Table S2. As in other crops, carrot hybrid varieties are generated by crossing two inbred parent lines, creating varieties with high observed heterozygosity (Maksylewicz & Baranski, 2013). Within each plot, we randomly selected three subplots each measuring 30 cm where soil measurements were made (see later). Within each subplot we randomly selected three plants on which trait and performance measurement were made. This resulted in 405 carrot plants sampled in a nested design with the following structure: nine farms, five plots within each farm (i.e. one for each variety), three subplots within each plot, and three individual plants within each subplot. See further details on planting procedures in Notes S1.

### Plant measurements

Plants were measured between 50 and 79 d after sowing, which coincides with the second phenological stage of carrot ontogeny (out of a typical 100–120 d harvest), an identified growth cycle stage to standardize sampling (Gonçalves *et al*., 2017). This date window corresponds with the published literature on measurements of gas exchange in carrot field trials (Kyei‐Boahen *et al*., 2003). Leaf physiological traits were measured using a portable gas analyzer and associated broadleaf chamber (Li‐Cor 6400 XT; Li‐Cor Biosciences, Lincoln, NE, USA). All measurements of light‐saturated photosynthesis (*A*_sat_, in μmol CO_2_ m^−2^ s^−1^), stomatal conductance (*g*_s_, in mmol m^−2^ s^−1^), and transpiration rate (TR, in mmol m^−2^ s^−1^) were taken on the youngest fully expanded intact leaf of each selected plant (i.e. one leaf per plant). Measurements were made between 07:00 h and 11:00 h at each farm site to avoid midday stomatal closure (Pérez‐Harguindeguy *et al*., 2013) with the following leaf chamber conditions: leaf temperature 20°C, irradiance 1200 μmol m^−2^ s^−1^, reference CO_2_ concentration 400 ppm, vapour pressure deficit < 2 kPa, and relative humidity 50–80%. Measurements were recorded after stabilization of flux values after clamping onto new leaves (approximately 60 s), and three measurements were taken for each leaf at 20 s intervals. An average of the three values was used in subsequent analysis to determine *A*_sat_, *g*_s_, and TR, which were calculated on a LA basis. Instantaneous photosynthetic WUE was calculated as the ratio of *A*_sat_ to TR (Seibt *et al*., 2008).

We measured leaf morphological traits on the same leaves as those used for Li‐Cor measurements. Immediately following gas exchange measurements, each leaf was removed and photographed separately. Diameter of all petioles (PD, in millimeters) at the top of the taproot was measured using electronic calipers. These leaves were placed into paper bags and transported to the laboratory for analysis. Leaf area (in cm^2^) was determined using image analysis in ImageJ v.1.45 software (Wayne Rasband, National Institute of Health, Bethesda, MD, USA). Leaves were shipped to the laboratory (University of Toronto Scarborough, Toronto, Canada), dried in the oven at 65°C for 48 h and then immediately weighed to obtain leaf dry mass (in grams), from which LMA (in g cm^−1^) was calculated.

Belowground morphological traits of each plant were measured on the same day of collection to avoid dehydration and shrinkage. Taproot length (RL, in centimeters) was measured as the distance from the crown to the tip of the storage root, defined here as having a diameter > 1 mm (McCormack *et al*., 2015; Turner *et al*., 2018). Taproot diameter (RD, in millimeters) was measured at the widest point of the taproot using electronic calipers. Taproots were placed in paper bags and transported to the laboratory for analysis. During transport, taproots were refrigerated to avoid moisture loss. Once in the laboratory, taproots were weighed to determine fresh mass (in grams) and dried to determine dry mass. We then calculated TTD as dry mass/volume (in g cm^−3^), where volume was estimated using length and diameter assuming a truncated‐cone shape, i.e. volume = 1/3 × π × RL × (RD^2 ^+ *r* ^2 ^+ RD × *r*), where *r* = 0.05 cm, which is the threshold radius we used to measure RL. Compared to a cylindrical shape approximation, the truncated‐cone approximation provided estimations of TTD with better statistical properties, namely lower skewness and kurtosis and no clear presence of outliers. Therefore, we conducted our analyses using TTD based on the truncated‐cone approximation, although we note that results were virtually unaffected by this decision.

### Soil measurements

After gas exchange measurements were completed at each site, soil samples were taken using a soil corer (111 cm^3^) adjacent to the taproot. Three soil cores were collected per variety per farm. Soil samples were placed in sealed plastic bags and transported to a laboratory at the University of Toronto Scarborough for analysis. To measure total soil N (SN, in mg g^−1^) and C (SC, in mg g^−1^) concentrations using a Leco CN628 (Leco Corp., St Joseph, MI, USA), the oven‐dried soil samples were ground using a Retsch Ball Mill (Retsch, Düsseldorf, Germany) and approximately 0.20 g of sample was weighed and placed into foil capsules. Samples were then analyzed on the Leco for soil N and soil C. To measure inorganic phosphorus levels (SP, in mg kg^−1^), a subsample of 2 g of sieved and air‐dried soil was placed in an Erlenmeyer flask and extracted with 20 ml of Bray 1. Samples were filtered using P5 filter paper into glass vials. Filtered samples were analyzed using a Lachat QuikChem 8500 Series 2 Flow Injection Analyzer (Lachat Instruments, Loveland, CO, USA) to measure inorganic P levels for each soil sample.

### Data analysis

#### Selection of traits and soil variables

Given the logistics of our field campaign, five physiological measurements were not possible within the optimal measurement timeframe. We therefore excluded these five plants from our dataset (representing *c*. 1% of the total sample size). Before analysis, traits and soil variables were transformed to improve normality and reduce the weight of extreme values, then standardized to zero mean and unit variance (see transformations in Tables S3, S4). After transformation and standardization, we identified and removed two outliers (extremely high LA values), leading to a total of 398 sampling units (plants) for subsequent analyses. We used pairwise correlations to select relatively independent subsets of traits and soil variables and so avoid collinearity among predictors. We considered that correlations above 0.6 may be problematic (see Dormann *et al*., 2013). All trait correlations were below 0.6 (Table S3), while soil N and C showed a correlation higher than 0.6 (Table S4). Because of the ecological importance of both soil N and C, we fit two alternative models (see later) featuring either of these variables and then compared the models in terms of Akaike information criterion (AIC).

#### Approach rationale

We sought to evaluate whether trait–environment relationships differed between carrot varieties based on the three‐dimensional approach. We used linear mixed models (LMMs) assuming Gaussian distribution of the response variable to model sqrt(*A*_sat_) and log(WUE) as a function of the selected traits and soil variables; we used separate models for sqrt(*A*_sat_) and log(WUE). We transformed *A*_sat_ and WUE to improve their normality. Before describing full multi‐trait and multi‐environment models, we outline our analysis for one trait (*T*) and one environmental variable (*E*), both standardized, influencing a generic performance variable *Y*. For simplicity, we first imagine that *Y* is measured on individual *i* in subplot *j* and we will ignore variety differences as well as plot and farm effects. The fixed‐effect part of this simplified model would be

(Eqn 1(a)) $$Y_{\mathrm{ij}}=\beta_{0}+\beta_{1}T_{i}+\beta_{2}T_{i}^{2}+\beta_{3}E_{j}+\beta_{4}E_{j}^{2}+\beta_{5}T_{i}E_{j}$$

where *β*_0_ is the *y*‐intercept of the fitted surface (Fig. S2). Since trait *T* is centered at zero, *β*_1_ measures the overall direction (positive or negative) and strength of trait *T_i_* value’s effect on plant performance (Aiken *et al*., 1991; Schielzeth, 2010), i.e. the mean slope of the trait–performance relationship (Rolhauser *et al*., 2021). Thus, *β*_2_ estimates the mean curvature of the trait–performance relationship. Negative values of *β*_2_ indicate ‘n’ shaped (optimum or unimodal) relationships, while positive values indicate ‘u’ shaped (bimodal) relationships. Removing environmental effects from Eqn 1(a) would give the classic quadratic model developed by Lande & Arnold (1983) to evaluate natural selection modes. Similarly, *β*_3_ and *β*_4_ reflect the mean slope and curvature of the environment–performance relationship (see Fig. S2). While doing this, we assess trait–environment relationships by estimating how the effect of *T* on *Y* depends on *E*. Rearranging Eqn 1(a) to gather terms for the mean (linear) effect of trait *T_i_* on *Y_ij_* yields:

(Eqn 1(b)) $$Y_{\mathrm{ij}}=\beta_{0}+T_{i}\left(\beta_{1}+\beta_{5}E_{j}\right)+\beta_{2}T_{i}^{2}+\beta_{3}E_{j}+\beta_{4}E_{j}^{2}$$

Then, the environment–dependent mean slope of trait–performance relationship is given by

(Eqn 2) $$\varphi_{j}=\beta_{1}+\beta_{5}E_{j}$$

(Fig. 1; Rolhauser *et al*., 2021). Thus, *β*_5_ measures the overall direction and strength of the trait–environment relationship (cf. Laughlin *et al*., 2018 where quadratic effects are omitted). Furthermore, *β*_1_ works as a *y*‐intercept for this relationship determining the ‘height’ of the line (Fig. 1).

#### Multi‐trait and multi‐environment models

We extended the earlier rationale to models including multiple traits and soil variables measured for several carrot varieties according to our hierarchical sampling design. We fitted initial multi‐trait and multi‐environment models that were then simplified through backward selection of the fixed‐effect part (i.e. no selection was applied to the random part). Since soil C and N were highly correlated, we first fitted two initial models for each response variable, one including soil N and one including soil C. Specifically, for both response variables, the initial, full models included four traits (LA, LMA, PD, and TTD), two soil variables (P and either N or C), and carrot variety (a categorical variable with five levels) as fixed effects. We also included as fixed effects all three‐way trait × soil × variety interactions (and all the associated nested two‐way interactions) as well as quadratic terms for all traits and soil variables. We included temperature and precipitation (measured at farm level) as fixed effect continuous covariates. We included farm, plot, and subplot as random‐effect nested intercepts (subplots nested within plots and these within farms). As a result, full models were built by 83 fixed effects and three random effects, which were estimated from the 398 observations. We fitted LMMs using the R‐package lme4 (Bates *et al*., 2015). We then performed backward selection on these initial models using the step function in the R‐package lmertest (Kuznetsova *et al*., 2017). This procedure eliminates the least significant term (*P*‐value > 0.05) following the principle of marginality; that is, lower order interactions that are contained in significant higher order interactions are kept in the model independently of their significance (Kuznetsova *et al*., 2015). The procedure stops when there are no nonsignificant terms to remove. We then compared the final models featuring either soil N or C based on AIC and selected the model with the smallest AIC. Diagnostic plots of the best models showed that residuals where fairly normal and that variance was reasonably homogenous across the range of fitted values (Figs S3, S4).

We calculated marginal and conditional *R* ^2^ (the proportion of the total variance explained by fixed effects and by both fixed and random effects, respectively) for the best models following the delta method (Nakagawa *et al*., 2017). We implemented *R* ^2^ calculations using the r.squaredGLMM function in the R‐package mumin (Barton, 2019). We evaluated the significance of model terms using type‐II analysis‐of‐variance tables based on the *F*‐statistic. Type‐II analysis‐of‐variance follows the principle of marginality, where each term is tested after all others in the same order or hierarchy, but ignoring the term’s higher‐order relatives (Fox & Weisberg, 2019). We calculated *F*‐statistics following Kenward–Roger method for the estimation of denominator degrees of freedom using the ANOVA function in the R‐package lmertest (Kuznetsova *et al*., 2017).

We also evaluated whether varieties differed in terms of the traits included in the *A*_sat_ and WUE models (LMA, LA, PD, TTD). This comparison will be useful to interpret the effects of varieties on *A*_sat_ and WUE; in particular, whether differences in physiological responses across varieties can be attributed to differences in trait variation. To this end, we used LMMs to model traits (one model for each trait) as a function of variety, as a fixed‐effect factor, and farm, plot, and subplot as random effects nested as in *A*_sat_ and WUE models.

## Results

### Photosynthetic rate

When explaining sqrt(*A*_sat_), backward selection from both soil C and soil N full models converged to a single best‐model solution. This reduced model (Tables 1, S5) used 15 parameters (12 fixed and three random effects) to explain sqrt(*A*_sat_) across 398 observations. The effect of farm (i.e. the estimated variance across farms) was larger than the effect of plot, and this was larger than the effect of subplot (Table S5). The marginal *R* ^2^ of this model was 0.130 with a conditional *R* ^2^ of 0.694.

**Table 1:** Type‐II analysis‐of‐variance table (following the principle of marginality) for the best linear mixed model (LMM) explaining instantaneous light‐saturated photosynthesis (*A*_sat_, sqrt transformed) of *Daucus carota* subsp. *sativus* (carrot) in nine farms across Canada.

Model term	Sum Sq	Mean Sq	Num df	Den df	*F* value	*P* value
Taproot tissue density (TTD)	0.6142	0.6142	1	349.8	3.145	0.0770
Soil phosphorus (P)	0.1490	0.1490	1	65.6	0.763	0.3856
Variety	1.3198	0.3300	4	28.1	1.689	0.1804
**TTD × soil P**	**1.4889**	**1.4889**	**1**	**370.5**	**7.623**	**0.0061**
**Soil P × Variety**	**3.3167**	**0.8292**	**4**	**38.8**	**4.245**	**0.0060**

The *F*‐statistics for model factors and variables are calculated based on Kenward–Roger degrees of freedom. Significant terms (*P* < 0.05) are shown in bold.

TTD and Soil P were log transformed prior to analysis.

**Fig. 2:**
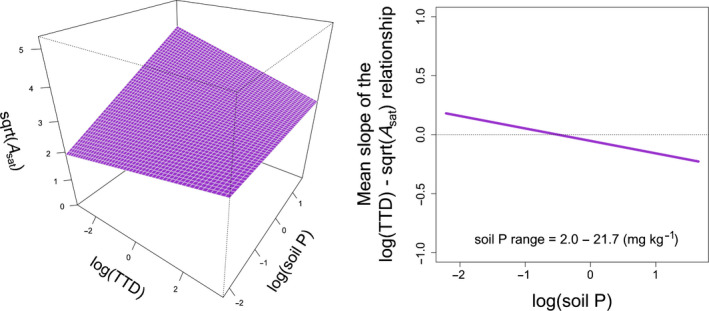
Instantaneous light‐saturated photosynthesis (*A*_sat_, sqrt transformed) of *Daucus carota* subsp. *sativus* (carrot) in nine farms across Canada as a function of the interaction between taproot tissue density (TTD, log transformed) and soil phosphorus (P) (log transformed) according to the model in Table 1. Both log(TTD) and log(soil P) were standardized to zero mean and unit variance. Left panel shows the response surface. Right panel shows the resulting trait–environment relationship, i.e. how trait effects on sqrt(*A*_sat_) change with log(soil P). According to Eqn 2, the main effect of TTD and the TTD × soil P interaction in Supporting Information Table S5 give the *y*‐intercept and the slope of this trait–environment relationship, respectively. The original range of soil P concentrations is shown within the panel. See Fig. S6 for surface plots with overlaid observations.

There was only one significant two‐way interaction between a trait and a soil variable (TTD × soil P) and one significant two‐way interaction with variety (soil P × Variety) (Table 1). The soil P × Variety interaction means that the slope of the relationship between *A*_sat_ and soil P changes across varieties. Specifically, there was a gradient of variation from positive soil P–*A*_sat_ relationships (most prominently for H1) to negative relationships (most prominently for OP3); the slope for H1 was significantly different from those for OP2 and OP3 (Fig. S5). The TTD × soil P interaction was negative, with standardized slope of −0.105 (Table S5). The resulting response surface was an asymmetrical saddle where *A*_sat_ is maximized by low‐TTD plants at high soil P (Fig. 2, left). The negative TTD × soil P interaction means that high‐TTD plants were favored in terms of maximized *A*_sat_ in soils with low P concentrations, whereas low‐TTD plants were favored at high P concentrations (Fig. 2, right).

### Water‐use efficiency

The best model derived from the full model including soil N outperformed the one derived from the full model including soil C (AIC values were 231.6 and 238.0, respectively). Therefore, we will hereafter focus on the results of the former. This reduced model (Tables 2, S6) used 45 parameters (42 fixed and three random effects) to explain log(WUE) across 398 observations. The effect of farm was larger than the effect of plot, and this was larger than the effect of subplot (Table S6). The marginal *R* ^2^ of this model was 0.134 with a conditional *R* ^2^ of 0.918. There were three significant three‐way interactions, all of them involving size traits LA and PD (LA × soil P × Variety, PD × soil P × Variety, PD × soil N × Variety), and two significant two‐way interactions which are nested within these three‐way interactions (PD × Variety and soil P × Variety) (Table 2). In particular, the soil P × Variety interaction means that the slope of the relationship between WUE and soil P changed across varieties. Specifically, the slope for H1 was significantly different from those for H2, OP2 and OP3 (Fig. S7). Importantly, the three‐way interactions mean that trait–environment relationships were not consistent across varieties.

**Table 2:** Type‐II analysis‐of‐variance table (following the principle of marginality) for the best linear mixed model (LMM) explaining instantaneous water‐use efficiency (WUE, log transformed) of *Daucus carota* subsp. *sativus* (carrot) in nine farms across Canada.

Model term	Sum Sq	Mean Sq	Num df	Den df	*F* value	*P* value
Leaf area (LA)	0.005	0.005	1	256.0	0.086	0.7696
Petiole diameter (PD)	0.002	0.002	1	347.2	0.026	0.8709
Soil phosphorus (P)	0.074	0.074	1	104.9	1.258	0.2646
Soil nitrogen (N)	0.019	0.019	1	96.5	0.318	0.5739
Variety	0.128	0.032	4	17.6	0.547	0.7038
Soil P^2^	0.283	0.283	1	108.4	4.848	0.0298
**Soil N^2^**	**0.420**	**0.420**	**1**	**116.8**	**7.184**	**0.0084**
LA **×** Soil P	0.029	0.029	1	280.5	0.490	0.4845
PD **×** Soil P	0.107	0.107	1	349.1	1.826	0.1775
PD **×** Soil N	0.002	0.002	1	325.4	0.036	0.8493
LA **×** Variety	0.111	0.028	4	140.3	0.473	0.7559
**PD × Variety**	**0.676**	**0.169**	**4**	**196.0**	**2.891**	**0.0235**
**Soil P × Variety**	**0.793**	**0.198**	**4**	**48.0**	**3.389**	**0.0160**
Soil N **×** Variety	0.343	0.086	4	50.0	1.464	0.2271
**LA × Soil P × Variety**	**0.826**	**0.207**	**4**	**176.2**	**3.531**	**0.0085**
**PD × Soil P × Variety**	**0.648**	**0.162**	**4**	**256.3**	**2.771**	**0.0278**
**PD × Soil N × Variety**	**0.966**	**0.241**	**4**	**152.0**	**4.128**	**0.0033**

The *F*‐statistics are calculated based on Kenward–Roger degrees of freedom. Significant terms (*P* < 0.05) are shown in bold.

LA and PD were square root transformed, while soil P and soil N were log transformed prior to analysis.

**Fig. 3:**
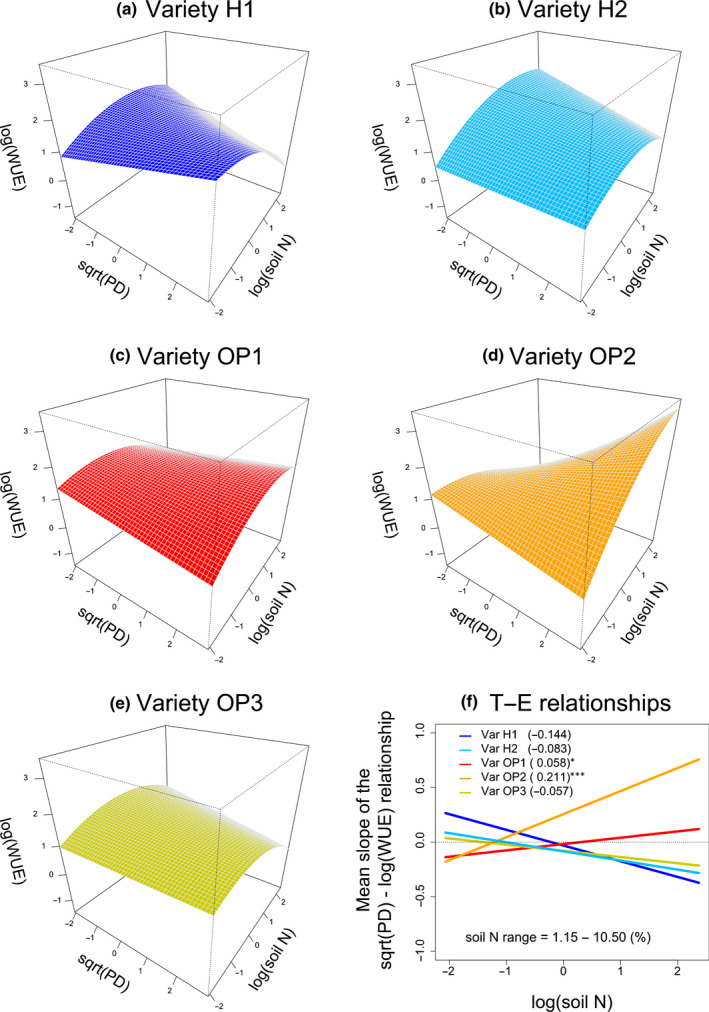
Instantaneous water‐use efficiency (WUE, log transformed) of *Daucus carota* subsp. *sativus* (carrot) in nine farms across Canada as a function of the three‐way interaction between petiole diameter (PD, sqrt transformed), soil nitrogen (N) (log transformed), and variety according to the model in Table 2. Both sqrt(PD) and log(soil N) were standardized to zero mean and unit variance. Panels (a) to (e) show response surfaces for each variety. Panel (f) shows the resulting trait–environment relationships, i.e. how sqrt(PD) effects on log(WUE) change with log(soil N) and across varieties. According to Eqn 2, the PD × Variety interactions and the PD × soil N × Variety interactions in Supporting Information Table S6 respectively give the *y*‐intercepts and the slopes for these trait–environment relationships. Numbers in parentheses are slope estimates and asterisks indicate significant differences with respect to variety H1. The original range of soil N concentrations is shown within the panel. See Fig. S8 for surface plots with overlaid observations.

The PD × soil N × Variety was the strongest three‐way interaction in terms of significance (Table 2). The slope of PD–soil N relationships (measured by the corresponding *β*_5_ parameter) differed across varieties and ranged between −0.144 (variety H1) to 0.211 (variety OP2) (Fig. 3; see also Table S6). Hybrid varieties (H1 and H2) showed negative PD–soil N relationships, whereas open‐pollinated varieties showed both positive (OP1 and OP2) and negative (OP3) relationships (Fig. 3). That is, wider petioles conferred higher WUE at low N for hybrid varieties (mostly notably for variety H1) but low WUE for two of three OP varieties (mostly notably for OP2). At high N, conversely, wider petioles conferred higher WUE for OP varieties OP1 and OP2 and lower WUE for both hybrid varieties.

**Fig. 4:**
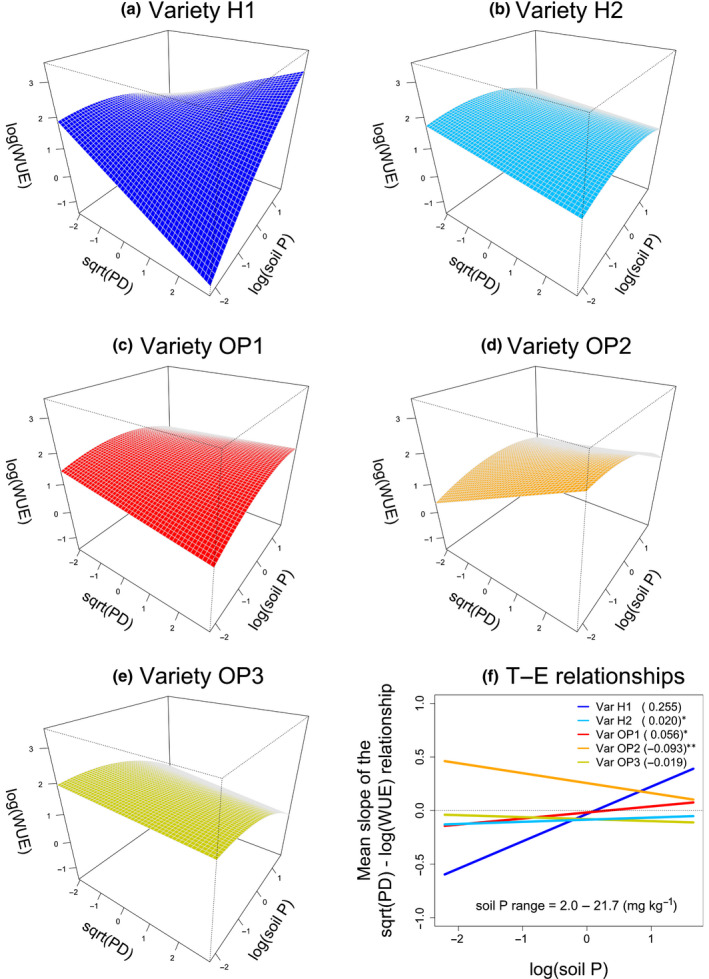
Instantaneous water‐use efficiency (WUE, log transformed) of *Daucus carota* subsp. *sativus* (carrot) in nine farms across Canada as a function of the three‐way interaction between petiole diameter (PD, sqrt transformed), soil phosphorus (P) (log transformed), and variety according to the model in Table 2. Both sqrt(PD) and log(soil P) were standardized to zero mean and unit variance. Panels (a–e) show response surfaces for each variety. Panel (f) shows the resulting trait–environment relationships, i.e. how sqrt(PD) effects on log(WUE) change with log(soil P) and across varieties. According to Eqn 2, the PD × Variety interactions and the PD × soil P × Variety interactions in Supporting Information Table S6 respectively give the *y*‐intercepts and the slopes for these trait–environment relationships. Numbers in parentheses are slope estimates and asterisks indicate significant differences with respect to variety H1. The original range of soil P concentrations is shown within the panel. See Fig. S9 for surface plots with overlaid observations.

Similarly, the PD × soil P × Variety interaction means that the slope of PD–soil P relationships differed across varieties (Fig. 4). We found the largest differences between H1 and OP2, showing positive and negative PD–soil P relationships, respectively (Fig. 4f). That is, wider petioles conferred higher WUE at low P for variety OP2, but low WUE for H1. At high P, wider petioles conferred lower WUE for OP2, while higher WUE for H1. The slope of H2 and OP1 were also significantly different from that of variety H1, although these PD–soil P relationships were relatively shallow (Fig. 4f).

**Fig. 5:**
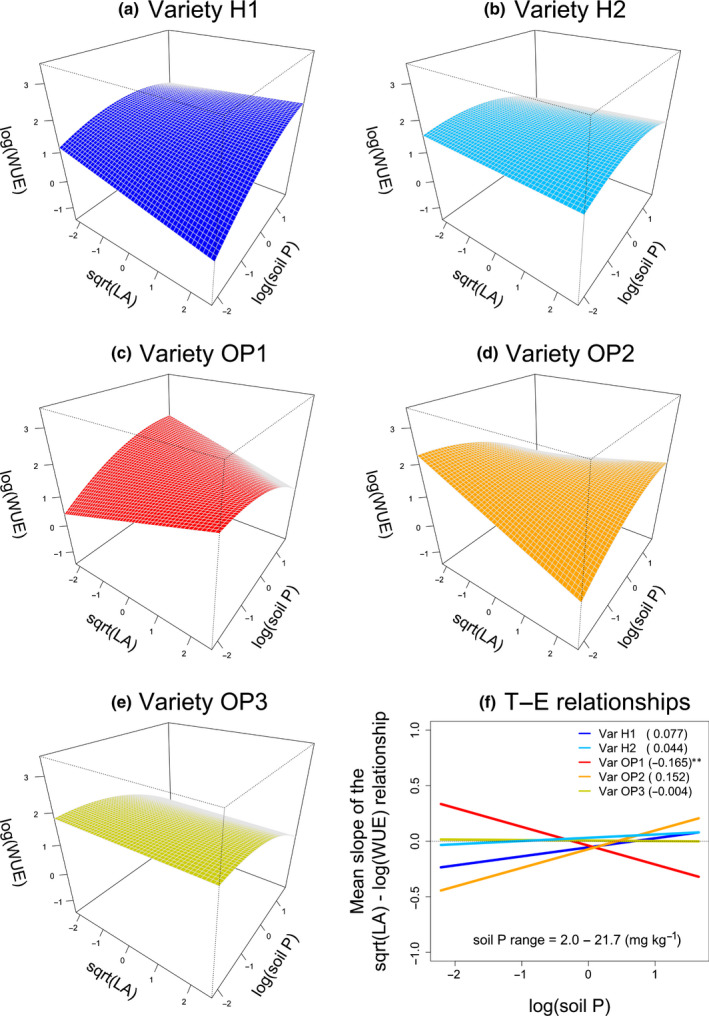
Instantaneous water‐use efficiency (WUE, log transformed) of *Daucus carota* subsp. *sativus* (carrot) in nine farms across Canada as a function of the three‐way interaction between leaf area (LA, sqrt transformed), soil phosphorus (P) (log transformed), and Variety according to the model in Table 2. Both sqrt(LA) and log(soil P) were standardized to zero mean and unit variance. Panels (a) to (e) show response surfaces for each variety. Panel (f) shows the resulting trait–environment relationships, i.e. how sqrt(LA) effects on log(WUE) change with log(soil P) and across varieties. According to Eqn 2, the LA × Variety interactions and the LA × soil P × Variety interactions in Supporting Information Table S6 respectively give the *y*‐intercepts and the slopes for these trait–environment relationships. Numbers in parentheses are slope estimates and asterisks indicate significant differences with respect to variety H1. The original range of soil P concentrations is shown within the panel. See Fig. S10 for surface plots with overlaid observations.

Fewer patterns were evident when looking at the LA × soil P × Variety interactions (Fig. 5), but varieties still differed greatly in their trait responses. Varieties OP2 and H1 showed the strongest positive LA–soil P relationships, whereas OP1 showed a clearly negative relationship; varieties H2 and OP3 showed shallower responses (Fig. 5). It is noteworthy that variety H1 and variety OP1 showed significantly different LA–soil P relationships (Fig. 5f). As a result of these interactions, larger leaves conferred higher WUE at low P for variety OP1, but low WUE for varieties H1 and OP2. Conversely, larger leaves conferred higher WUE at high P for varieties H1 and OP2, while low WUE for OP1.

Overall, the most salient differences between varieties were between H1 and OP2 in two trait–environment relationships, PD–soil N (the most important factor affecting WUE) and PD–soil P (Figs 3f, 4f). Hybrid H1 showed an overall positive WUE response to soil P (Fig. S7) and predicted WUE maxima at both high soil P and low soil N mediated by high PD (Figs 3a, 4a, respectively). In contrast, open‐pollinated OP2 showed an overall negative WUE response to soil P (Fig. S7) and predicted WUE maxima at both low soil P and high soil N mediated by high PD (Figs 3d, 4d, respectively). Altogether, these results indicate that the maximization of WUE by these varieties was favored by contrasting soil conditions, high P and low N for H1 and low P and high N for OP2.

## Discussion

### Multiple dimensionalities of performance, trait, and environment interactions

Plant traits, soil variables, variety genotypes, and their interactions explained around 13% of the variation in light‐saturated photosynthesis (*A*_sat_) and WUE. Random effects explained considerably more variation, 56% for *A*_sat_ and 78% for WUE, respectively for *A*_sat_ and WUE. These results illustrate that individually measured traits can provide at least a partial explanation of these complex physiological responses, even within a single species. These trait–environment responses can be interpreted as the result of phenotypic plasticity within genotypes. Western open pollinated carrot varieties showed considerable within‐variety genetic variation despite their relative phenotypic stability (Stelmach *et al*., 2021), so further research is warranted on the relative contributions of phenotypic plasticity and genetic effects on within‐species trait–environment relationships.

Many studies have shown negative relationships between LMA and *A*_sat_ both across species (e.g. Wright *et al*., 2004; Poorter & Bongers, 2006) and within crops (e.g. Martin *et al*., 2018). Surprisingly, however, LMA did not appear as an important trait for *A*_sat_ in our study. Instead, TTD appeared to be the sole relevant morphologic trait explaining carrot *A*_sat_. In our dataset, pairwise trait correlations were mild (Table 1), suggesting that nonsignificant leaf effects on *A*_sat_ were not an artifact of collinearity. Rather, these results suggest that carrot *A*_sat_ was more linked with belowground carbon storage than with leaf anatomy. This result is consistent with the domestication history of carrot, in which increased root size was key to the divergence of the cultivated subspecies from its wild relatives (Ellison, 2019). In contrast, effects of LMA on WUE appear to be less clear in the literature with studies showing strong negative correlations (e.g. Craufurd *et al*., 1999, peanut) to studies showing negative but nonsignificant effects, both in crops (Anyia & Herzog, 2004, cowpea) and across forests species (Poorter & Bongers, 2006). Our results concur with the latter as we found no effect of LMA on WUE, which instead depended on the other leaf traits analyzed here, LA and PD.

### Variety–independent trait–environment relationships

We found trait responses that were consistent across varieties, and these involved interactive effects of TTD on *A*_sat_. TTD was related to *A*_sat_ through a negative interaction with soil P, whereas the main effect of TTD on *A*_sat_ was nonsignificant. In general, anatomical structures which protect root functioning (e.g. stele and cell‐wall proportions) result in high tissue density and constrain plant growth (Wahl & Ryser, 2000). As a result, denser carrots are usually tougher and more durable. In our experiment, flimsier (less dense) taproots (lower TTD) were favored in terms of maximized *A*_sat_ in high‐P soils, whereas tougher taproots were favored in low‐P soils. These results agree with trait economic theory (Reich, 2014) and previous empirical work (Kramer‐Walter *et al*., 2016; Butterfield *et al*., 2017) in that constrained soil conditions promote denser, likely more durable root tissues. More generally, the change in the slope of TTD–*A*_sat_ from positive to negative as soil P increases suggests a soil‐dependent physiological coordination between belowground organs and performance measured aboveground. Interestingly, the absence of a TTD × soil P × Variety interaction in our analysis suggests that such physiological coordination is relatively fixed at the level of species.

Our correlative approach does not allow us to determine the causal link between TTD, soil P, and *A*_sat_. Presumably, causal relationships between TTD and performance could be bidirectional. On the one hand, TTD variation may be a result of differences in photosynthesis, since allocation to roots, and the determination of TTD, necessarily occurs after photosynthates are generated in leaves. On the other hand, altered root structures can modify fibrous root architecture, which may be critical for photosynthesis via water acquisition and stomatal conductance (Isaac *et al*., 2021).

### The role of crop varieties in trait–environment relationships

The main effect of the factor ‘variety’ was nonsignificant in both *A*_sat_ and WUE models, indicating that genetic effects only manifested through interactions with other variables. That is, carrot varieties did not directly affect *A*_sat_ or WUE but, instead, modulated the effects of traits, soil variables, and their interactions. We did not find significant differences (alpha = 0.05) between varieties along any of the traits included in the *A*_sat_ and WUE models (LMA, LA, PD, TTD) (Table S7). This means that varieties display fairly similar ranges of trait variation, and that the differences across varieties discussed later may not be attributed to differences in trait variation but to differences in trait effects on performance. Overall, while trait–environment effects on *A*_sat_ were consistent across varieties, our analysis of WUE points out that trait–environment relationships can vary noticeably within a single species. The remainder of this section therefore focuses on WUE responses.

Differences between varieties were most notable between H1 and OP2, which appeared to thrive in contrasting soil conditions. For H1, wider petioles maximized WUE at low N and high P, whereas wider petioles maximized WUE at high N and low P for OP2. These results show that different macronutrient combinations, rather than high vs low overall soil fertility, were associated with wider petioles for different varieties. In addition, varieties H1 and OP2 – with deeply curved response surfaces – show phenotypic variation that facilitates variability in WUE over a soil nutrient gradient, while varieties with shallow responses (most notably, H2 and OP3) show more stability in both phenotype and WUE across environments. While it would be premature to recommend specific varieties for certain soil conditions based on a single study, these results demonstrate the capacity of the three‐dimensional framework to identify crop varieties that optimize performance under specific environmental conditions or show stability across a range of conditions, both important priorities for organic and PPB.

Causes of cross‐variety differences in our study were not clear, however. *A priori*, one major source of variation could be the type of variety, hybrid vs open‐pollinated. In general, hybrids are thought to be more resilient to environmental stresses via heterosis or hybrid vigor (Goff, 2011). One might then predict that hybrid varieties would show shallower responses to environmental gradients compared to open‐pollinated varieties. However, our estimates of trait–environment relationship strength (*β*_5_) showed that both H1 and OP2 displayed relatively strong responses, while other hybrid and OP varieties also displayed shallow responses. It appears that differences in heterosis associated with variety types did not seem to drive variation in the strength of trait–environment relationships in our experiment. Another important source of variation across our varieties could be divergent breeding environments, ranging from a single Pacific Northwest organic farm to multiple conventionally managed sites in Continental Europe (Table S2). Unfortunately, we lack precise environmental and agronomic information on the breeding and seed production conditions for these cultivars, and pedigree information is not available for all cultivars. Future studies could investigate how breeding environments affect varieties’ performance responses to trait–environment interactions. For PPB programs, it would be of particular importance to compare performance of varieties bred in organic systems and those bred in conventional systems with synthetic inputs (Atlin *et al*., 2001; Murphy *et al*., 2007; Isaac *et al*., 2021).

### Implications for breeding programs

Historically, plant breeding programs (mostly focused on high‐input systems) have characterized the interactive effects of crop genotypes (G) and environmental conditions (E) on phenotypic traits of agronomic interest (Atlin *et al*., 2001; Crespo‐Herrera & Ortiz, 2015). That is, phenotypic traits (e.g. yield; foliage characteristics) are considered as response variables affected by the G × E interaction. This approach contrasts with ours, which was inspired by research in other areas interested in the responses of organisms to environmental variation, namely natural selection applied to trait‐based community ecology (Shipley, 2010; Laughlin & Messier, 2015; Shipley *et al*., 2016). In our approach, phenotypic characteristics that reflect individual performance (e.g. yield, *A*_sat_ or WUE) are conceived as a function of other phenotypic characteristics ideally related to the eco‐physiological interaction of individuals with the environment (e.g. leaf size). We provide a novel way to investigate the performance of varieties selected in particular environments (e.g. conventional, organic, regional) on organic farms and identify trait–environment combinations that might illuminate mechanisms for any differential performance. Functional traits important for performance in organic environments could be further associated with genomic data, facilitating genomic prediction to select parental material for breeding projects (Corak, 2021). The three‐dimensional functional trait approach to plant breeding presented in this work – grounded by farmer input through PPB frameworks and potentially informed by genomic data – presents new opportunities for eco‐evolutionary crop development.

Integrating a PPB framework with this functional trait approach offers opportunities to increase its practical utility for farmers seeking varietal improvements, particularly in response to environmental conditions affected by climate variability (Isaac & Martin, 2019). To investigate the impact of performance variables at the farm scale, future on‐farm research could test associations with performance variables directly targeted by farmers, such as flavor (possibly related to TTD), harvestability (possibly related to PD), or stand establishment (possibly related to LA). In particular, flavor has emerged as a primary breeding priority for the organic and consumer‐direct marketplace (Colley, 2021), so it would be critical to understand associations between functional traits and flavor compounds, perceived flavor attributes, or hedonic liking. For example, firm carrot texture has been identified as a negative sensory attribute (Seljåsen *et al*., 2012), but it is unknown whether perceived firmness is associated with TTD. If such an association exists, our results would suggest that selecting for *A*_sat_ (likely via yield selection) in low‐P soils could result in dense taproots with poor consumer acceptance. If differences in TTD are not perceptible as differences in firmness, breeding programs would have more freedom to select for performance variables without sacrificing sensory quality.

### Implications for basic ecology

Functional traits relate to environmental gradients via fundamental eco‐physiological principles that should lead to general trait–environment relationships (Westoby *et al*., 2002; Garnier *et al*., 2016). However, our results showed clear differences in trait–environment relationships across genotypes (varieties) within a single species. To our knowledge, this is the first formal test of intraspecific variation in trait–environment relationships based on the three‐dimensional performance–trait–environment approach. This evidence adds to that already found across species and communities (Westoby *et al*., 2002; Kichenin *et al*., 2013; Lajoie & Vellend, 2015; Garnier *et al*., 2016; Funk *et al*., 2017; Dong *et al*., 2020; Buchanan *et al*., 2021; Sarker *et al*., 2021). Future research could therefore focus on elucidating the origin of this heterogeneity. For instance, exploring how a given trait affects another trait’s interaction with the environment, which constitutes an alternative perspective to the study of trait–trait–environment interactions focused on how the environment modulates trait interactions (Laughlin & Messier, 2015).

### Conclusions

As we expected, we found a negative relationship between soil P availability and TTD (an economic trait inversely related to plant acquisitiveness) when performance was measured as *A*_sat_. However, we found a much more complex picture when performance was measured as instantaneous WUE, with varieties following our general prediction in some instances (e.g. a positive PD–soil P for H1) but having an opposite response in others (e.g. a negative PD–soil N for H1). Some standardized slopes of these WUE‐based trait–soil relationships were more than twice as steep as the *A*_sat_‐based, variety‐independent TTD–soil P slope, although some varieties, typically H2 and OP3, showed much shallower responses. These results underscore the complex multidimensionality of trait–environment relationships and highlight the need for further research on the relative contributions of phenotypic plasticity, genetic effects, and breeding environments on within‐crop trait–environment relationships.

Increased productivity and resilience to climate extremes in the organic and low‐input vegetable farming sector can be achieved through improvements in regional organic vegetable variety breeding efforts. However, there remains a critical need for breeding paradigms that explicitly focus on a better understanding of relationships between crop functional trait expression under various environmental constraints in order to maximize crop performance and nutrient acquisition strategies. Here we show novel analytical tools that explicitly recognize the three‐dimensional structure involving crop performance, crop trait, and environmental axes. We derived these tools inspired by ideas generated in trait‐based community ecology. We uncover the multidimensionality of trait–environment relationships within a crop, with key implications for a more nuanced understanding of the role of breeding on crop performance in heterogeneous environments.

## Author contributions

AGR and MEI conceived the ideas; EW and MEI designed the experiment; EW collected the data; AGR analyzed the data and led the writing, with significant contributions from SH, HW, CT, AL and MEI.

## Supporting information


**Fig. S1** Map of participating farms.
**Fig. S2** Visualizations of model predictions.
**Fig. S3** Residual plots of the best photosynthesis (*A*_sat_) model.
**Fig. S4** Residual plots of the best water‐use efficiency (WUE) model.
**Fig. S5** Interaction effects between variety and soil P on *A*_sat_.
**Fig. S6** Visualization of data points in Fig. 2.
**Fig. S7** Interaction effects between variety and soil P on WUE.
**Fig. S8** Visualization of data points in Fig. 3.
**Fig. S9** Visualization of data points in Fig. 4.
**Fig. S10** Visualization of data points in Fig. 5.
**Notes S1** Details of planting procedures in farms.
**Table S1** Description of participating farms.
**Table S2** Description of selected varieties.
**Table S3** Pearson correlations between plant traits.
**Table S4** Pearson correlations between soil variables.
**Table S5** Summary of photosynthesis (*A*_sat_) model.
**Table S6** Summary of water‐use efficiency (WUE) model.
**Table S7** Trait differences across varieties.
Please note: Wiley Blackwell are not responsible for the content or functionality of any Supporting Information supplied by the authors. Any queries (other than missing material) should be directed to the *New Phytologist* Central Office.

## Data Availability

Data available from the Dryad Digital Repository, doi: 10.5061/dryad.tdz08kq27 (Isaac *et al*., 2022).
