# Supplementary material for: A trait–environment relationship approach to participatory plant breeding for organic agriculture
Source: New Phytol. 2022 May 24;235(3):1018–31. doi: 10.1111/nph.18203 (PMC9322327; doi:10.1111/nph.18203)
Supplement: Supplementary file 1 — Fig. S1 Map of participating farms. Fig. S2 Visualizations of model predictions. Fig. S3 Residual plots of the best photosynthesis (A sat) model. Fig. S4 Residual plots of the best water‐use efficiency (WUE) model. Fig. S5 Interaction effects between variety and soil P on A sat. Fig. S6 Visualization of data points in Fig. 2. Fig. S7 Interaction effects between variety and soil P on WUE. Fig. S8 Visualization of data points in Fig. 3. Fig. S9 Visualization of data points in Fig. 4. Fig. S10 Visualization of data points in Fig. 5. Notes S1 Details of planting procedures in farms. Table S1 Description of participating farms. Table S2 Description of selected varieties. Table S3 Pearson correlations between plant traits. Table S4 Pearson correlations between soil variables. Table S5 Summary of photosynthesis (A sat) model. Table S6 Summary of water‐use efficiency (WUE) model. Table S7 Trait differences across varieties. Please note: Wiley Blackwell are not responsible for the content or functionality of any Supporting Information supplied by the authors. Any queries (other than missing material) should be directed to the New Phytologist Central Office. [file NPH-235-1018-s001.pdf]

New Phytologist Supporting Information

Article title: A trait–environment relationship approach to participatory plant breeding for organic agriculture

Authors: Andres G. Rolhauser, Emma Windfeld, Solveig Hanson, Hannah Wittman, Chris Thoreau, Alexandra Lyon, and Marney E. Isaac

Article acceptance date: 23 April 2022.

The following Supporting Information is available for this article:

Figure S1. Map of Canada displaying the 9 farms that participated in this study. The inset maps of British Columbia and Ontario display the 5 and 4 farms in each region, respectively. Images from Google Earth.

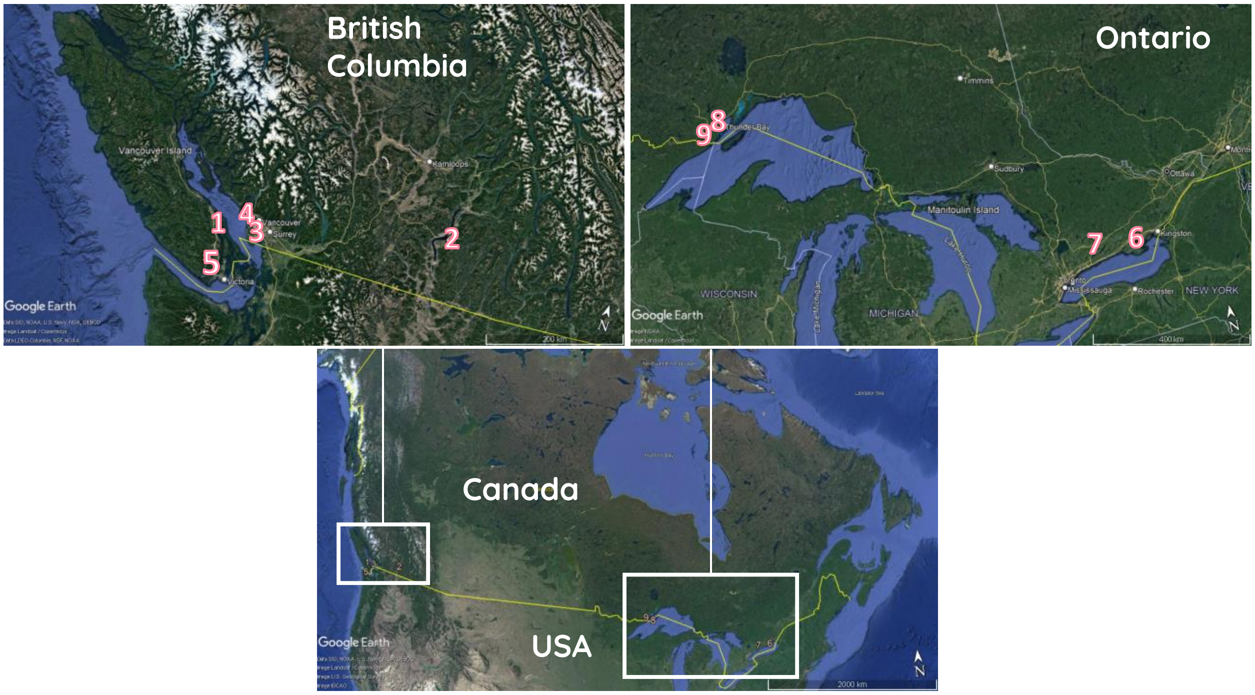

Table S1. 1981 to 2010 Canadian Climate Normals data for average temperature, average precipitation, annual growing degree days (GDD), number of annual frost-free days, soil class, soil textural group. Mean annual temperature (MAT) and precipitation (MAP) values were calculated from 30-year historic averages for June–August. GDD were calculated by taking the average of the daily maximum and minimum temperatures minus the base temperature for carrot (5°C).

**Farm**	**MAT** (ºC)	**MAP** (mm)	**GGD** (ºC)	**Frost-free** (days)	**Soil class**	**Soil texture**
1	17.3	97.2	2094	195	Podzol	Sandy loam
2	18.4	115.2	1989	140	Gleysol	Sandy loam
3	17.2	126.1	2177	237	Gleysol	Loam
4	17.2	126.1	2177	237	Brunisol	Sandy loam
5	16.6	178.7	1640	134	Brunisol	Sandy loam
6	20.5	215.1	2329	171	Luvisol	Loam
7	18.3	227.5	1916	132	Brunisol	Loam
8	16.6	237.9	1425	98	Luvisol	Clay
9	16.6	237.9	1425	98	Luvisol	Clay

Table S2. Information on the five carrot varieties selected for this study.

**Code**	**Name**	**Source**	**Type**	**Regional selection**	**Breeder**	**Breeding location(s)**	**Breeding conditions**	**Approx. breeding latitude**	**Intro-duction Date**
H1	Bolero	Johnny's	Hybrid	Broad	Vilmorin	Unknown	Conventional	Unknown	1989
H2	Naval	High Mowing	Hybrid	Broad	Bejo	South Europe (seed line) and Netherlands (pollen line)	Conventional	~44 N and 52N	2005
OP1	Dolciva	High Mowing	Open pollinated	Eastern	Sativa	Switzerland	Organic and Biodynamic	~46 N	2017
OP2	Nash's Nantes	BC EcoSeed Co-op	Open pollinated	Western	Nash Huber	Sequim, WA	Organic	48 N	<2000
OP3	Rumba	BC EcoSeed Co-op	Open pollinated	Western	Nash Huber	Sequim, WA	Organic	48 N	2001

Notes S1. Details of planting procedures in farms

Seed packets, management instructions, and record-keeping instructions were mailed to participating farmers prior to the 2019 growing season. Farmers were instructed to sow seeds 2-3 weeks prior to the last frost date in their region, depending on weather conditions and farmers' normal planting dates. Seeding dates ranged from 13 June to 26 June 2019. All information regarding seeding was submitted via the online platform *SeedLinked.* Farmers planted 2 row-feet (0.6m) of each variety with no smaller than 25 cm between-row spacing. Seeding rate was standardized as the contents of one entire seed package per variety, as the number of seeds in each packet had been adjusted according to the germination rate. One farmer chose to use a seeder to seed at 1 inch (2.54 cm) spacing; carrot was hand-seeded at all other sites. Farmers could use row covers to prevent pest damage; farmers at 2 sites chose to use white fabric row covers. Thinning was not mandatory; if they chose to do so, farmers could thin carrot to 2.5cm. Farmers at 2 sites thinned carrot plants.

Table S3. Pearson correlations between four plant traits: leaf area (LA), leaf mass per area (LMA), petiole diameter (PD), and taproot tissue density (TTD). Traits were transformed to improve normality and reduce the weight of extreme values. Sqrt: square root; log: natural logarithm. All correlations are <0.6.

	**sqrt(LA)**	**sqrt(LMA)**	**sqrt(PD)**	**log(TTD)**
**sqrt(LA)**	1			
**sqrt(LMA)**	-0.132	1		
**sqrt(PD)**	0.340	0.406	1	
**log(TTD)**	0.060	-0.019	-0.427	1

Table S4. Pearson correlations between four soil variables: total nitrogen (N), total carbon (C) and available phosphorus (P) contents. Soil variables were transformed to improve normality and reduce the weight of extreme values. log: natural logarithm. Correlations >0.6 are shown in orange.

	**log(P)**	**log(C)**	**log(N)**
**log(P)**	1		
**log(C)**	0.400	1	
**log(N)**	0.542	0.857	1

Figure S2. Fixed-effect structure of our linear mixed model (LMM) used to explain variation in the performance of individual *i* at site *j* (Y_{ij}; see equation at the top). Y_{ij} (grey surface) is modeled as function of trait *T* (e.g., leaf size) and environmental variable *E* (e.g., soil N), both standardized to zero mean and unit variance. Parameters β_1 and β_2 are the linear and quadratic effects of *T* on *Y*; β_3 and β_4 are the linear and quadratic effects of *E* on *Y*; and β_5 is the effect of *E* on *T* (see Approach rationale in the main text). This model reproduces unimodal trait–performance (orange and blue) and environment–performance (green and red) relationships. In this example, the result is a negative *T*–*E* relationship, which evidences as a ridge growing from the low-*T* and high-*E* corner to the high-*T* and low-*E* corner (for this, β_5 was set to -1, whereas β_2 and β_4 were both set to -0.5, and the remaining parameters to zero). Dotted lines are modeled relationships at different *T* or *E* levels while continuous lines are their counterparts projected onto 2-D planes.

$$Y_{ij} = exp\left(\beta_0 + \beta_1 T_i + \beta_2 T_i^2 + \beta_3 E_j + \beta_4 E_i^2 + \beta_5 T_i E_j\right)$$

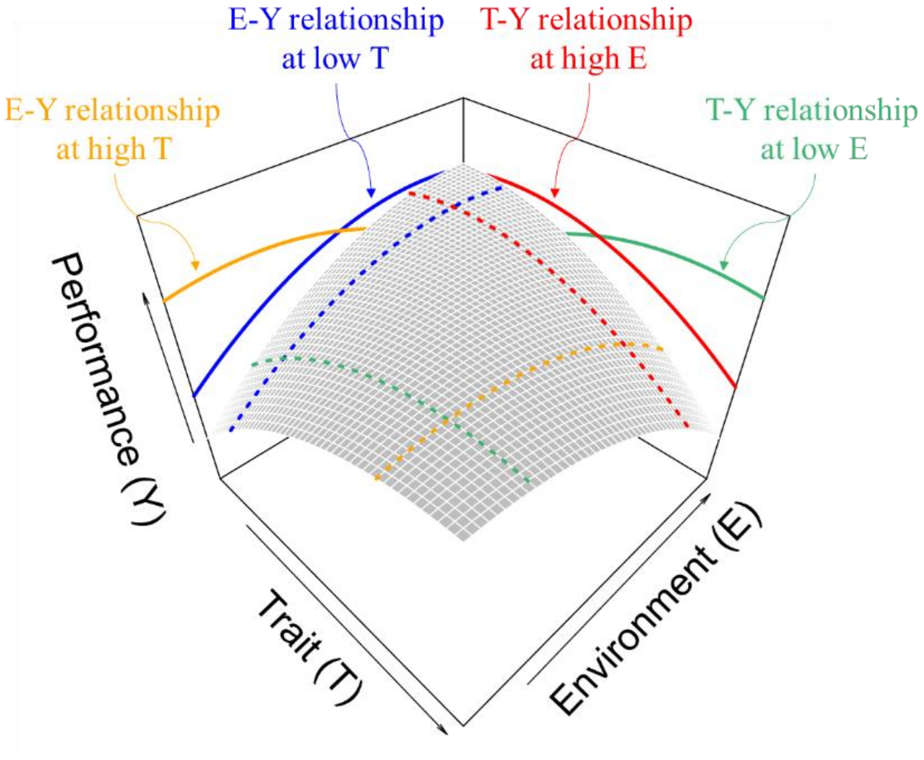

Figure S3. Residual plots of the best LMM explaining instantaneous light-saturated photosynthesis (A_{sat}, sqrt transformed) of *Daucus carota* subsp. *sativus* (carrot) in 9 farms across Canada. See model details in Tables 1 (main text) and S5.

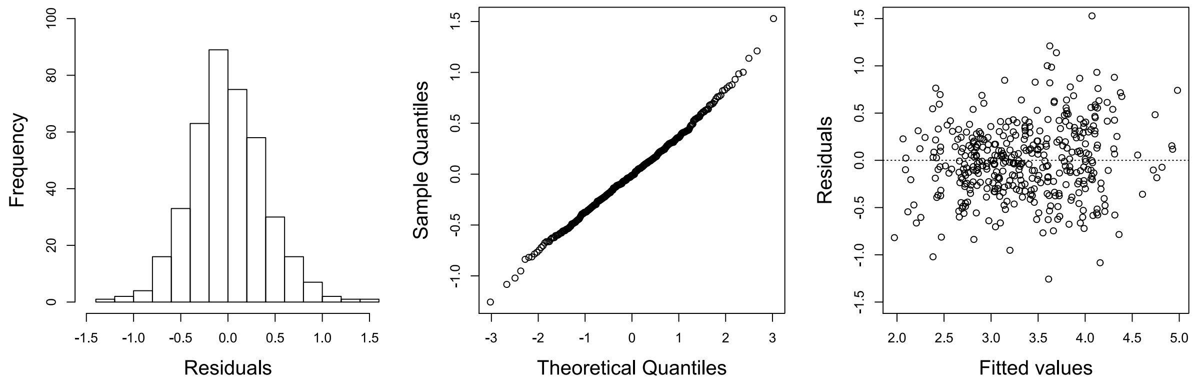

Figure S4. Residual plots of the best LMM explaining instantaneous water-use efficiency (WUE, log transformed) of *Daucus carota* subsp. *sativus* (carrot) in 9 farms across Canada. See model details in Tables 2 (main text) and S6.

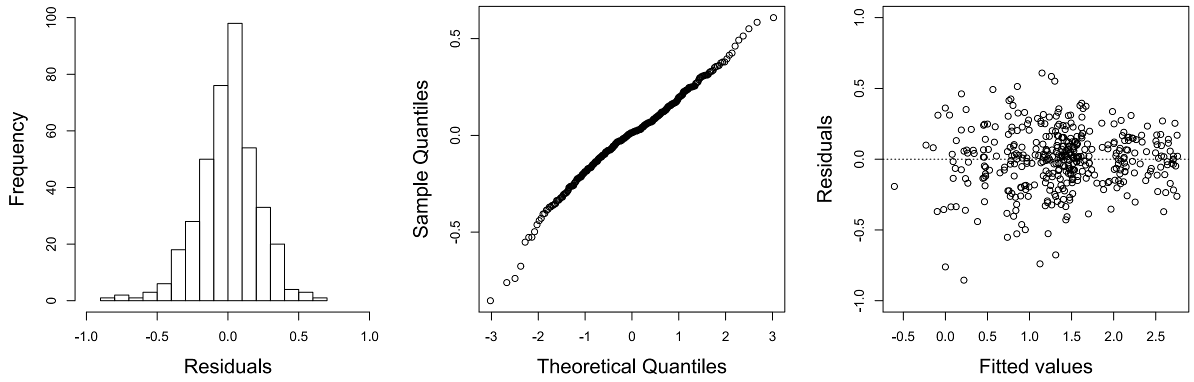

Table S5. Summary tables for the best LMM explaining instantaneous light-saturated photosynthesis (A_{sat}, sqrt transformed) of *Daucus carota* subsp. *sativus* (carrot) in 9 farms across Canada. For the analysis, traits and soil variables were transformed as shown in Tables S3 and S4. Codes for traits and soil variables as in Tables S3 and S4.

```
Random effects:
 Groups              Name        Variance Std.Dev.
 sub-plot:(plot:farm) (Intercept) 0.06252  0.2500
 plot:farm            (Intercept) 0.08168  0.2858
 farm                 (Intercept) 0.21133  0.4597
 Residual                         0.19532  0.4420
Number of obs: 398, groups:  sub-plot:(plot:farm), 134; plot:farm, 45; farm, 9

Fixed effects:
             Estimate Std. Error        df t value Pr(>|t|)
(Intercept)   3.18860    0.19381  15.22851  16.452 0.0000000000414 ***
TTD          -0.05273    0.03706 346.47453  -1.423        0.155748
P             0.32378    0.12755  60.22744   2.538        0.013740 *
varOP1        0.22314    0.16771  27.30326   1.330        0.194365
varOP3        0.31486    0.17151  28.46882   1.836        0.076858 .
varOP2        0.05183    0.16704  26.80002   0.310        0.758739
varH2         0.18676    0.16739  27.20007   1.116        0.274304
TTD:P        -0.10553    0.03786 370.59522  -2.788        0.005583 **
P:varOP1     -0.24207    0.15328  37.64869  -1.579        0.122651
P:varOP3     -0.72943    0.17950  34.24632  -4.064        0.000267 ***
P:varOP2     -0.35967    0.15535  34.58503  -2.315        0.026654 *
P:varH2      -0.23408    0.14507  39.44171  -1.614        0.114589
---
Signif. codes:  0 '***' 0.001 '**' 0.01 '*' 0.05 '.' 0.1 ' ' 1
```

Figure S5. Estimates of interaction effects between *Daucus carota* subsp. *sativus* (carrot) variety and soil P on A_{sat} according to the model in Table S5. Estimates measure the slope of the response of A_{sat} to soil P variation. Asterisks indicate significant differences with respect to variety H1 (*: p<0.05; ***: p<0.001); see Table S5.

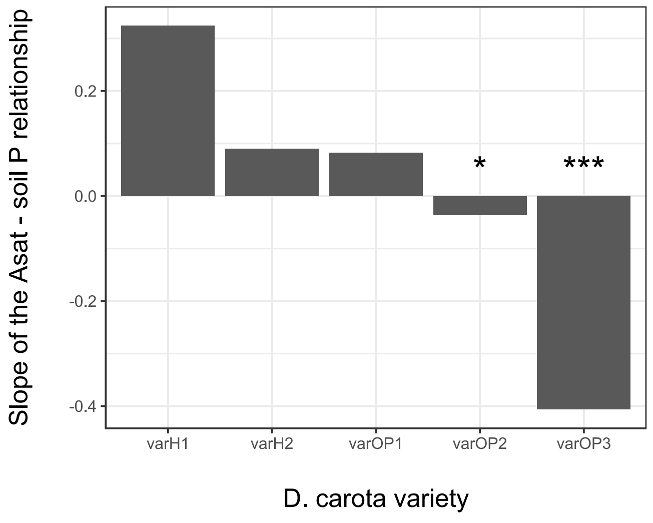

Figure S6. Visualization of the response of instantaneous light-saturated photosynthesis (A_{sat}) (sqrt transformed) to the interaction between log(TTD) and log(Soil P) according to the model in Table 1. Idem 3d plot in **Figure 2**, except that the observations are plotted, and the plot is rotated.

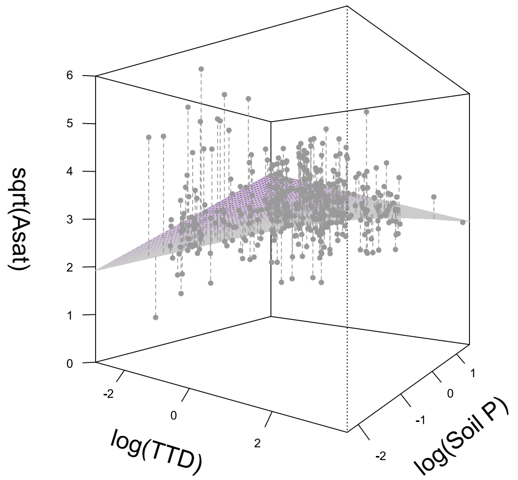

Table S6. Summary tables for the best LMM explaining instantaneous water-use efficiency (WUE, log transformed) of *Daucus carota* subsp. *sativus* (carrot) in 9 farms across Canada. Due to the parametrization used (Bates *et al.*, 2015), interaction estimates are calculated as differences from a base level, by default (as in here), the one that comes first in the alphabet. For instance, the slope of the PD–Soil P relationship for variety H1 (i.e., β_5 for this variety, see equation 1) is estimated by parameter PD:P, while the slope corresponding to variety H2 is estimated by the sum of PD:P and PD:P:varH2. The same applies to *y*-intercepts (β_1 in equation 1), estimated by PD in the case of variety 1 and by PD + PD:varH2 for variety H2. For the analysis, traits and soil variables were transformed as shown in Tables S3 and S4. Codes for traits and soil variables as in Tables S3 and S4.

Random effects:

Groups	Name	Variance	Std.Dev.
subplot:(plot:farm)	(Intercept)	0.01524	0.1235
plot:farm	(Intercept)	0.06096	0.2469
farm	(Intercept)	0.48321	0.6951
Residual		0.05846	0.2418

Number of obs: 398, groups: subplot:(plot:farm), 134; plot:farm, 45; farm, 9

Fixed effects:

	Estimate	Std. Error	df	t value	Pr(>|t|)	
(Intercept)	1.6929445	0.2614958	11.3734255	6.474	0.0000393	***
LA	-0.0554052	0.0749750	176.1168798	-0.739	0.460901	
PD	-0.0316199	0.0740602	249.8362337	-0.427	0.669784	
P	0.2213440	0.1118937	70.7115468	1.978	0.051807	.
N	-0.1061841	0.1354146	68.4076173	-0.784	0.435665	
var9	-0.0208483	0.1386617	21.1876977	-0.150	0.881908	
var14	-0.1598120	0.1432398	24.2285011	-1.116	0.275496	
var19	-0.1922616	0.1348813	19.9714097	-1.425	0.169480	
var21	0.0001258	0.1379010	21.7248699	0.001	0.999280	
I(P^2)	-0.1140285	0.0503126	103.9839498	-2.266	0.025497	*
I(N^2)	-0.1244713	0.0450827	110.9816260	-2.761	0.006745	**
LA:P	0.0813750	0.0567927	235.6953735	1.433	0.153228	
PD:P	0.2552934	0.0797634	318.2553308	3.201	0.001510	**
PD:N	-0.1435652	0.0760223	250.3661110	-1.888	0.060120	.
LA:var9	0.0151642	0.0958199	138.0941345	0.158	0.874485	
LA:var14	0.0610213	0.0944680	109.4770060	0.646	0.519666	
LA:var19	-0.0166407	0.0941793	170.1541103	-0.177	0.859960	
LA:var21	0.0861369	0.0994429	193.2680846	0.866	0.387458	
PD:var9	0.0138086	0.0878098	205.3310053	0.157	0.875198	
PD:var14	-0.0483622	0.0986040	194.1194591	-0.490	0.624356	
PD:var19	0.2879713	0.0954003	227.3827270	3.019	0.002829	**
PD:var21	-0.0535172	0.0967959	251.8960733	-0.553	0.580831	
P:var9	-0.2372103	0.1335784	42.1093584	-1.776	0.082991	.
P:var14	-0.6247212	0.1773174	42.3482569	-3.523	0.001037	**
P:var19	-0.3788742	0.1334470	46.9602655	-2.839	0.006664	**
P:var21	-0.3718828	0.1235843	51.1592381	-3.009	0.004059	**
N:var9	0.1433182	0.1433514	51.9504625	1.000	0.322057	
N:var14	0.0848970	0.1895870	51.1924136	0.448	0.656187	
N:var19	0.1940811	0.1423328	54.6005062	1.364	0.178298	
N:var21	0.3480555	0.1387875	53.9356616	2.508	0.015189	*
LA:P:var9	-0.2508715	0.0844538	174.9560909	-2.971	0.003391	**
LA:P:var14	-0.0858521	0.1005723	142.3169078	-0.854	0.394741	
LA:P:var19	0.0867507	0.0861735	199.8719216	1.007	0.315297	
LA:P:var21	-0.0521143	0.0834808	198.0020499	-0.624	0.533171	
PD:P:var9	-0.1988500	0.0951229	257.7010107	-2.090	0.037557	*
PD:P:var14	-0.2738648	0.1454137	217.9570331	-1.883	0.060985	.
PD:P:var19	-0.3481535	0.1053268	300.0169307	-3.305	0.001063	**
PD:P:var21	-0.2353011	0.1015764	322.6270924	-2.316	0.021157	*
PD:N:var9	0.2014702	0.1002908	137.5610241	2.009	0.046509	*
PD:N:var14	0.0867838	0.1228093	150.0983135	0.707	0.480877	
PD:N:var19	0.3544374	0.0996029	185.1048218	3.559	0.000474	***
PD:N:var21	0.0601861	0.0924681	199.5255309	0.651	0.515869	

Signif. codes: 0 '***' 0.001 '**' 0.01 '*' 0.05 '.' 0.1 ' ' 1

Figure S7. Estimates of interaction effects between *Daucus carota* subsp. *sativus* (carrot) variety and soil P on WUE according to the model in Table S6. Estimates measure the slope of the response of A_{sat} to soil P variation. Asterisks indicate significant differences with respect to variety H1 (**: p<0.01); see Table S6.

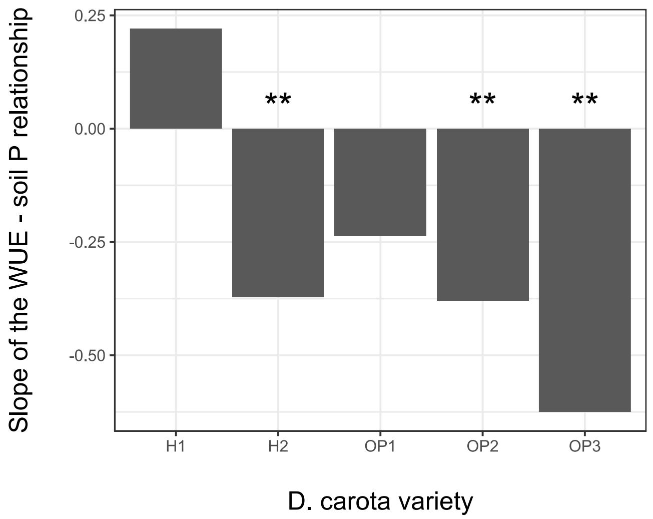

Figure S8. Visualization of the response of instantaneous water use efficiency (WUE) to the three-way interaction between sqrt(PD), log(Soil N), and variety according to the model in Table 2. Idem 3d plots in **Figure 3**, except that the observations are plotted, and the plots are rotated.

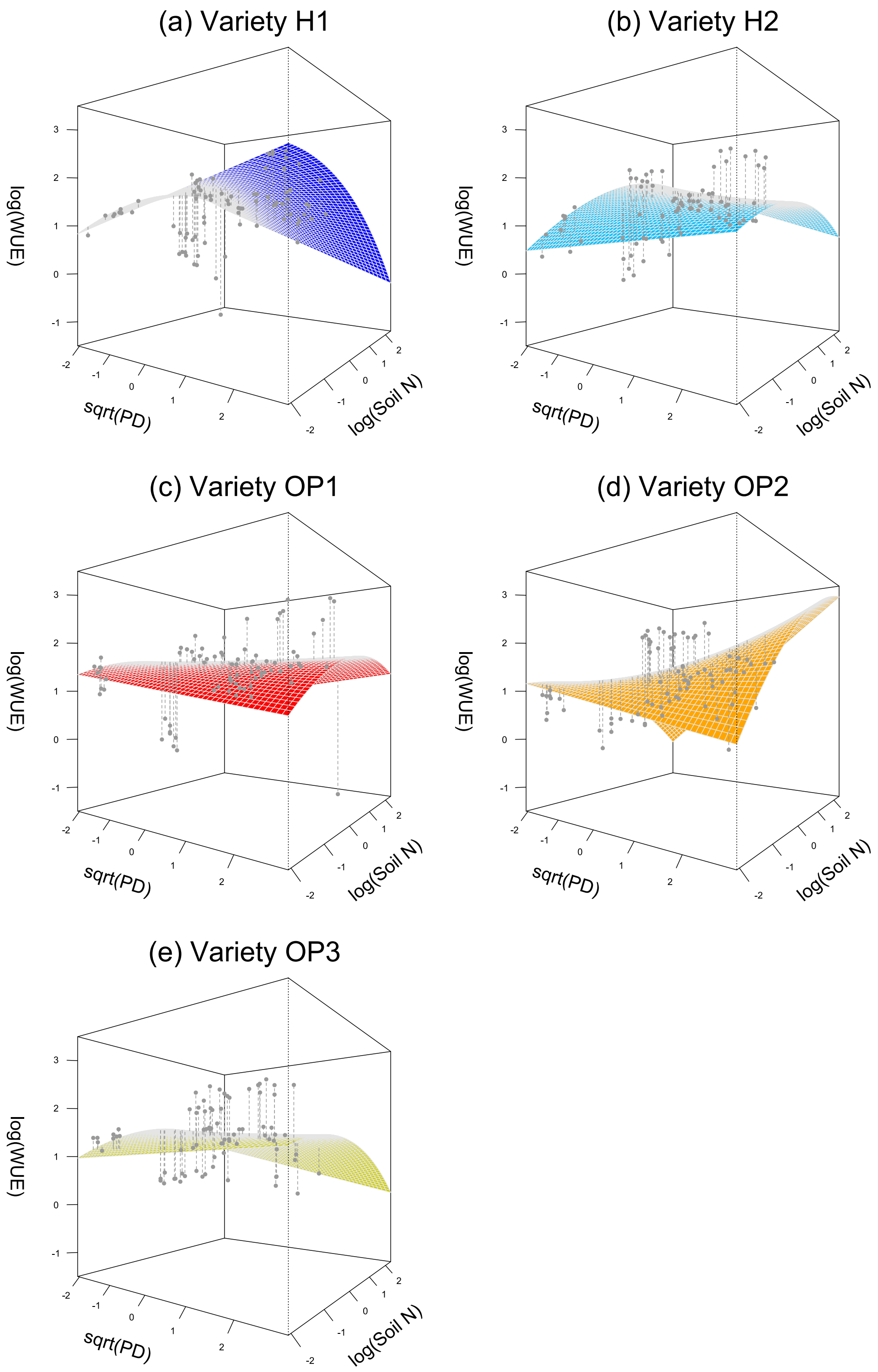

Figure S9. Visualization of the response of instantaneous water use efficiency (WUE) to the three-way interaction between sqrt(PD), log(Soil P), and variety according to the model in Table 2. Idem 3d plots in **Figure 4**, except that the observations are plotted, and the plots are rotated.

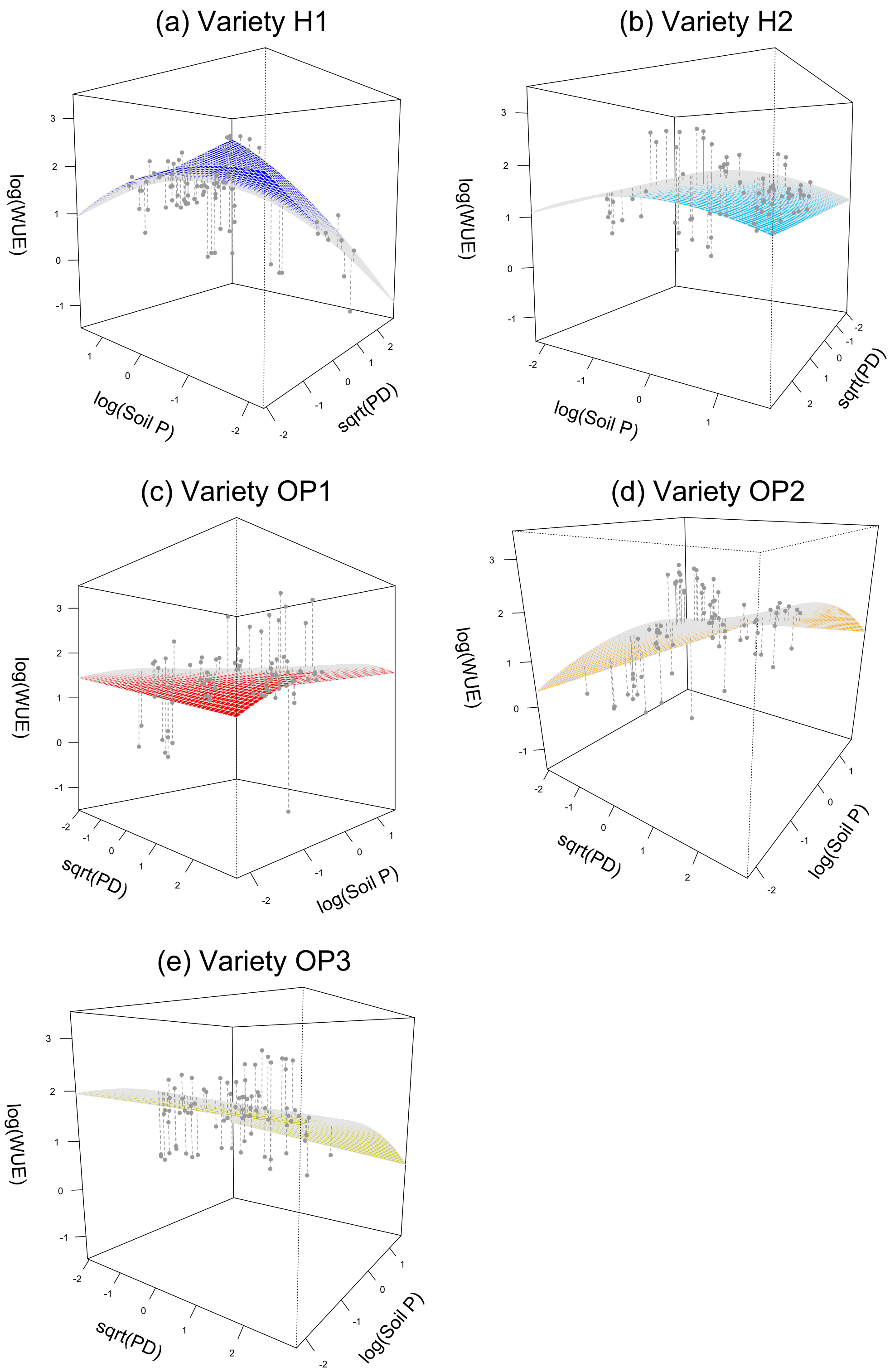

Figure S10. Visualization of the response of instantaneous water use efficiency (WUE) to the three-way interaction between sqrt(LA), log(Soil P), and variety according to the model in Table 2. Idem 3d plots in **Figure 5**, except that the observations are plotted, and the plots are rotated.

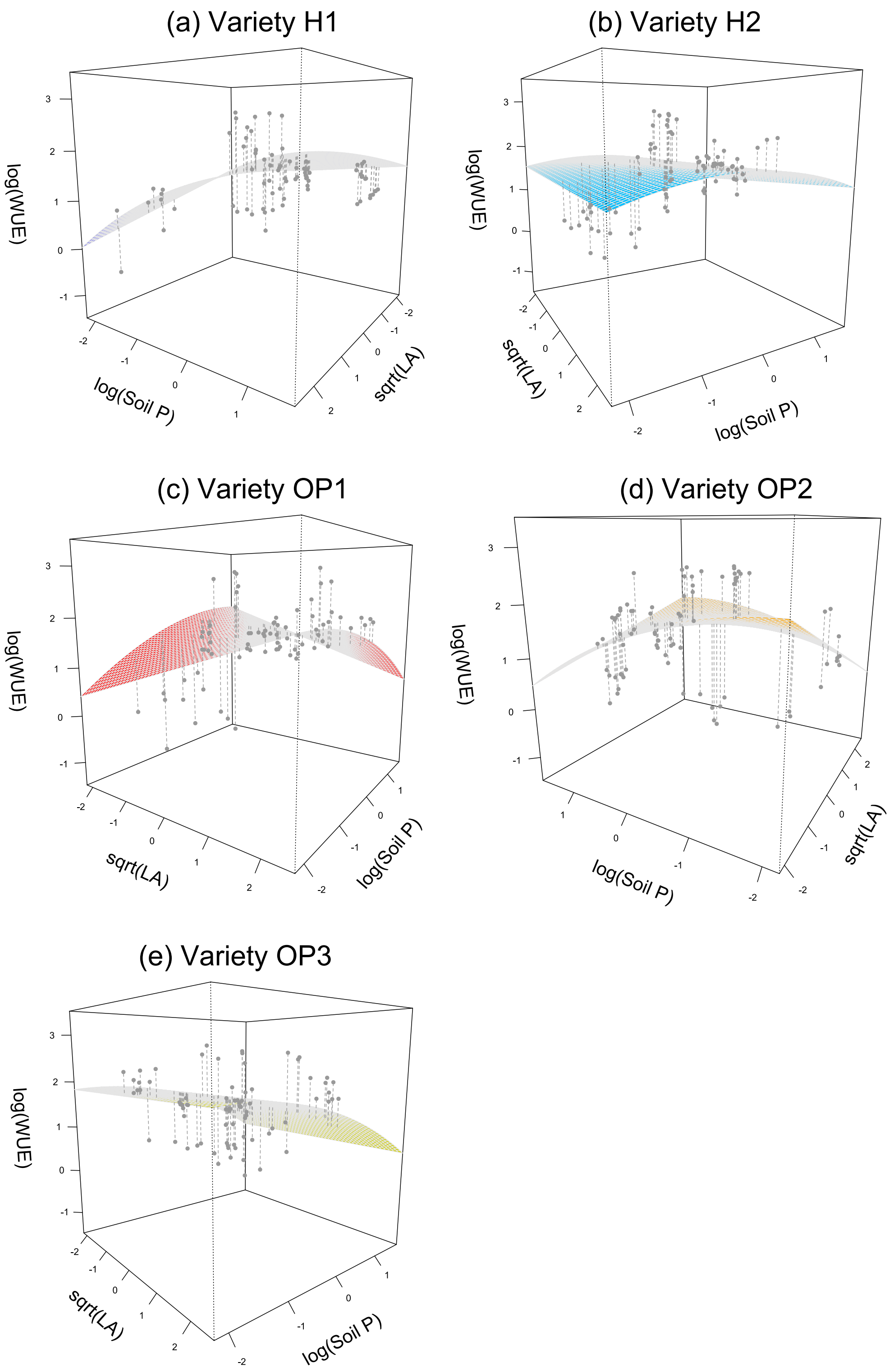

Table S7. Effects of *Daucus carota* subsp. *sativus* (carrot) variety on the four traits included in A_{sat} and WUE models. One model was separately fit to each trait. Each model contained a (transformed) trait as the sole fixed effect, and farm, plot, and subplot as nested random-effect (subplots nested within plots and these within farms). *F*-statistics are calculated based on Kenward-Roger degrees of freedom.

**Trait**	**Sum Sq**	**Mean Sq**	**Num DF**	**Den DF**	***F* value**	***P* value**
sqrt(LA)	0.426	0.107	4	31.99	0.726	0.581
sqrt(LMA)	0.844	0.211	4	31.99	0.637	0.640
sqrt(PD)	2.098	0.524	4	31.98	2.397	0.071
log(TTD)	1.011	0.253	4	31.99	0.664	0.622

References

Bates D, Maechler M, Bolker B, Walker S. **2015**. Fitting Linear Mixed-Effects Models Using lme4. *Journal of Statistical Software* **67**: 1–48.
